# Molecular mechanisms of viral oncogenesis in haematological malignancies: perspectives from metabolic reprogramming, epigenetic regulation and immune microenvironment remodeling

**DOI:** 10.1186/s40164-025-00655-2

**Published:** 2025-05-10

**Authors:** Qing Xiao, Yi Liu, Xuejiao Shu, Ya Li, Xiaomei Zhang, Chaoyu Wang, Sanxiu He, Jun Li, Tingting Li, Tingting Liu, Yao Liu

**Affiliations:** Department of Hematology-Oncology, Chongqing Key Laboratory for the Mechanism and Intervention of Cancer Metastasis, Chongqing University Cancer Hospital, Chongqing, 400030 China

**Keywords:** Oncogenic viruses, Haematological malignancies, Metabolism, Epigenetic regulation, Immune microenvironment

## Abstract

Haematological malignancies are one of the most common tumors, with a rising incidence noted over recent decades. Viral infections play significant roles in the pathogenesis of these malignancies globally. This review delves into the contributions of various known viruses—specifically Epstein-Barr virus (EBV), human immunodeficiency virus (HIV), human T-cell leukemia virus type 1 (HTLV-1), Kaposi’s sarcoma-associated herpesvirus (KSHV), human cytomegalovirus (HCMV), hepatitis B virus (HBV), hepatitis C virus (HCV), and human papillomavirus (HPV)—in the development of haematological malignancies. These viruses are shown to drive tumorigenesis through mechanisms, such as metabolic reprogramming, epigenetic modifications, and remodeling of the immune microenvironment. By directly disrupting fundamental cellular functions and altering metabolic and epigenetic pathways, these viruses foster an immune milieu that supports both viral persistence and tumor growth. A thorough understanding of these viral oncogenic processes is crucial not only for etiological discovery but also for developing targeted interventions. This review emphasizes the need for continued research into the specific ways these viruses manipulate the host cell’s metabolic and epigenetic environments, aiming to provide insights that could guide future advancements in treatment modalities.

## Introduction

Hematological malignancies are a group of diseases caused by the abnormal proliferation of malignant cells in the blood or lymphatic tissues, including many types of leukemias, lymphomas and plasma cell diseases. Recent studies have shown that viral infection is one of the important factors in the development of malignancies. Viruses may initiate cancer through interactions with specific proteins and thrive when the immune system is compromised [1]. The International Agency for Research on Cancer (IARC) recognizes several oncogenic viruses as human carcinogens [2, 3], including Epstein-Barr virus (EBV), Kaposi’s sarcoma-associated herpesvirus (KSHV), human T-cell leukemia virus type 1 (HTLV-1), hepatitis B virus (HBV), hepatitis C virus (HCV), and human papilloma virus (HPV) are classified as carcinogenic to humans [2]. Merkel cell polyomavirus (MCV) is classified as possibly carcinogenic to humans [3]. Other viruses can cause cancer indirectly, such as human immunodeficiency virus (HIV), whose indirect carcinogenicity involves a state of chronic inflammation and immunosuppression caused by infected cells, making them more susceptible to infection with a number of oncogenic viruses and making it more difficult to clear these viruses [4, 5].

When a cell is infected by a virus, the virus can alter the metabolism of the host cell via diverse mechanisms to support its replication and proliferation. This metabolic reprogramming not only supplies the virus with essential energy and biosynthetic precursors, but also affects normal cellular functions and drive oncogenesis [6–8]. Meanwhile, viral oncoproteins can affect cellular gene expression by modifying host DNA methylation, triggering chromatin reorganization, and producing virally produced non-coding RNAs [9]. Thus, epigenetic changes resulting from viral infection become a key factor in the promotion of oncogenic features, such as uncontrolled cell proliferation, evasion of normal cell death mechanisms, and promotion of angiogenesis, among other cancer features. At the same time, these viruses significantly shape the metabolic programs of immune cells, directly or indirectly inducing remodeling of the immune microenvironment and promoting an immunosuppressive tumor microenvironment to evade immune surveillance [10]. Recent studies have found that hematologic malignancies exhibit abnormal cellular metabolism, such as increased glycolysis, altered amino acid metabolism, and reprogramming of lipid metabolism. In addition, recurrent methylation-associated mutations, aberrant DNA methylation profiles, and dysregulated expression of histone deacetylases were also demonstrated, especially in leukemias and lymphomas. Therefore, a comprehensive exploration of the dynamics of the cellular metabolic reprogramming, epigenetic mediators and immune microenvironmental status in hematological oncogenesis will facilitate the development of innovative targeted therapies to improve the treatment response and prognosis of patients.

## EBV

EBV, also known as human herpesvirus 4 (HHV-4), was the first human oncogenic virus to be discovered. EBV infection is primarily transmitted through saliva, but can also be transmitted through sexual contact and organ transplants. There is a strong correlation between EBV infection and several types of B-cell lymphomas [11], including endemic/sporadic Burkitt lymphoma (eBL/sBL), diffuse large B-cell lymphoma (DLBCL), classical Hodgkin’s lymphoma (cHL), primary central nervous system lymphoma (PCNSL), post-transplant lymphoproliferative disorder (PTLD), primary effusion lymphoma (PEL), and plasmablastic lymphoma (PBL) [12, 13].

### Viral proteins and infection mechanisms

**Fig. 1:**
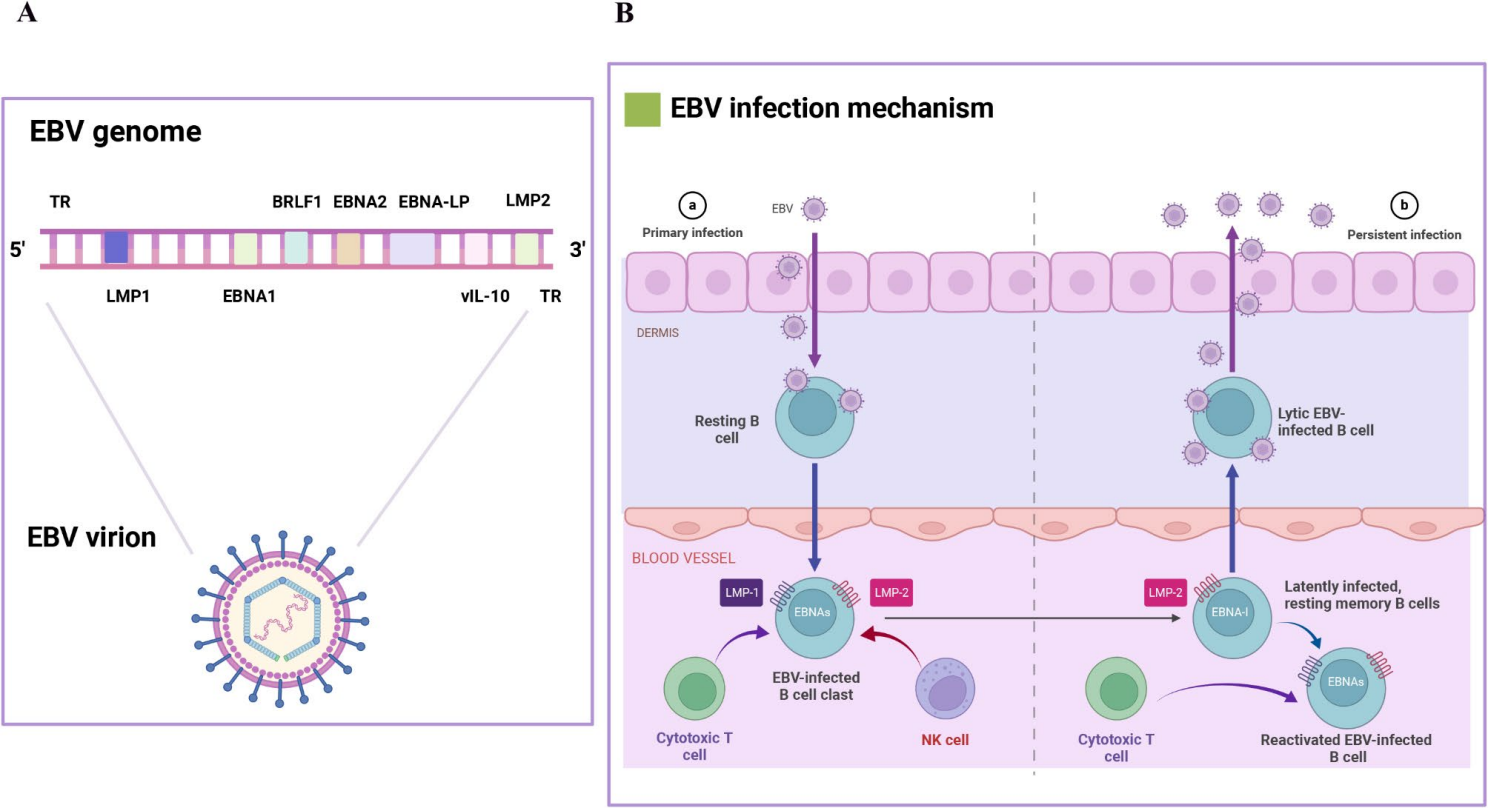
EBV-encoded latent proteins and infection mechanisms. (**A**) EBV-infected cells express several latent antigens, including EBV nuclear antigens and latent membrane proteins. (**B**) EBV infections are classified as latent or lytic. EBV can periodically transition to the lytic cycle, leading to viral replication, shedding and subsequent dissemination. This figure was created with BioRender.com

The target cells of EBV are B lymphocytes and epithelial cells, and its mechanism of entering host cells is identical in many respects to that of other members of the herpesvirus family [14–18]. EBV-infected cells express several latent antigens, including EBV nuclear antigens (EBNA1, EBNA2, EBNA3A, EBNA3B, EBNA3C and EBNA-LP) and latent membrane proteins (LMPs, including LMP1, LMP2A and LMP2B). These EBV-derived antigens activate resting B cells to produce proliferating lymphoblasts and provide survival signals to maintain infected cells [19]. EBV infections are classified as latent or lytic. During latent infection, there are four modes, latent 0, I, II, III, based on how these genes are expressed in different combinations in EBV-infected cells (Table 1), which are characterized by a gradual restriction of the viral gene expression pattern to evade immune surveillance. Eventually, EBV establishes a persistent residency in memory B cells characterized by a lack of viral antigen expression (latency 0), thereby evading T cell recognition and acting as a viral reservoir. EBV can periodically transition to the lytic cycle, leading to viral replication, shedding and subsequent dissemination, which is involved in B cell transformation and tumorigenesis [20–23] (Fig. 1).

**Table 1:** EBV latency patterns

	Latency			
	0	I	II	III
EBERs	+	+	+	+
EBNA1	-	+	+	+
EBNA2	-	-	-	+
EBNA3A, 3B, 3 C	-	-	-	+
EBNA-LP	-	-	-	+
LMP1	-	-	+	+
LMP2A, 2B	-	-	+	+

EBER, EBV-encoded small RNAs; EBNA, EBV nuclear antigen; EBNA-LP, EBV nuclear antigen-leader protein; LMP, latent membrane protein

### EBV infection acts as a tumorigenic factor through metabolic reprogramming

#### Glucose metabolism

Metabolic reprogramming is required to meet anabolic demands when B cells are subjected to EBV or receptor-driven proliferative attack [24–26]. Indeed, EBV significantly reprograms host cell metabolism, upregulating oxidative phosphorylation and glycolysis [27]. During EBV immortalization of B cells into lymphoblastoid cell lines (LCLs), the increased energy requirements for sustained proliferation lead to upregulation of glycolysis and extracellular acidification rates [24, 28–30]. EBV plays an important role in activating NF-κB signaling, which supports the proliferation and resistance of cancer cells by increasing glucose input [31, 32]. Indeed, in EBV-transformed B cells, inhibition of the NF-κB pathway reduces glucose uptake, thereby triggering their autophagy [29]. Meanwhile, the increase of hypoxia-inducible factor 1α (HIF-1α) in EBV-infected B cells varies across latency programs: LMP1 stimulates the transcriptional activity of HIF-1α through the p42/44 MAPK pathway and contributed to its stabilization [33, 34]; both LMP1 and EBNA1 upregulated HIF-1α and its downstream targets IL-8 and VEGF [35]; EBNA3s expressed in lymphoid chronic leukemia bind prolyl hydroxylases (PHD1 and PHD2) and block their phosphorylation activity, thereby stabilizing HIF-1α [36].

#### Lipid metabolism

In vitro, EBV transforms quiescent B cells into immortalized lymphoblastoid cells through a series of viral latency programs [37, 38]. The EBV transformation program activates lipid metabolism, transforming B cells into immortalized LCLs [39]. Benjamin E Gewurz et al. found that cholesterol and fatty acid biosynthesis pathways are among the most abundant pathways that are induced by EBV. EBNA2, sterol regulatory element binding proteins (SREBP), and MYC each have essential roles in the induction of cholesterol and fatty acid pathways [24]. Ai Kotani et al. found that secreted phospholipase A2 (sPLA2) drove hydrolysis of tumor-derived extracellular vesicles (EVs), increased lipid mediator production, altered the behavior and function of EVs, and skewed macrophages toward a tumor-associated macrophage-like (TAM) phenotype, which facilitated the EBV-induced development of B-cell lymphoma [40].

A key gene associated with dysregulated carbohydrate and lipid metabolism in lymphomas is MYC [35]. MYC is a well-known oncogene that promotes tumorigenesis by regulating key genes associated with patterns that promote cell growth and proliferation [41]. MYC may promote the fatty acid synthase (FASN) and the acetyl coenzyme A cellular supply, which are required for lipid synthesis and nuclear histone acetylation [42]. Interestingly, aberrant expression of MYC was found in EBV-infected lymphoma cells, both in BL and other lymphomas [43]. The concomitant occurrence of EBV infection and aberrant expression of MYC may indicate that they either act synergistically or compensate for each other in driving lymphomagenesis. For example, in EBV-infected LCLs, EBNA2 plays a role in lymphoid chronic leukemia proliferation and survival through the EBV super-enhancer [43].

#### Amino acid metabolism

EBV highly remodels metabolic pathways in newly infected B cells [24, 27, 44–46]. EBV upregulates plasma membrane methionine transporters, particularly those critical for methionine import [24]. Similarly, the metabolic master regulator MYC is highly expressed in BL, which increases levels of methionine import and metabolism [47, 48]. Benjamin E. Gewurz et al. found that EBV-infected B cells are highly sensitive to perturbation of extracellular methionine or serine concentrations, or to the blockage of the one-carbon metabolic cycle of methionine and folate. Disruption of methyl flow from methionine to the EBV epigenome inhibits Burkitt B cell latency I state. Methionine restriction (MR) de-represses EBV latent membrane proteins and the abortive lytic cycle, consisting mainly of highly immunogenic immediate early antigens. MR also disrupts the EBV-mediated transformation of primary B cell [49].

Dihydroorotate dehydrogenase (DHODH) and de novo pyrimidine synthesis are highly induced by EBV in newly infected B cells [50]. The pathway of B cell stimulation or transformation determines specific roles for cysteine import, glutathione synthesis and GPX4 synthesis. Solute carrier family 7 member 11 (SLC7A11) is already upregulated 2 days after EBV infection and progressively reaches higher levels later in the physiological transition of cells to lymphoblastoid cells [45, 51]. Cells normally import cystine via the SLC7A11/xCT transporter and then reduce it to cysteine or synthesize cysteine via the methionine metabolic transsulfuration pathway [52]. Thus, EBV may have an increased requirement for cystine import via SLC7A11, especially during Burkitt-like hyperproliferation, where the amino acid biosynthetic pathway may be maximally induced [39].

#### One-carbon metabolism

Metabolic stress is a major obstacle to EBV-mediated B-cell transformation [28]. EBV-encoded EBNA2 and its target MYC are required for the up-regulation of the central mitochondrial one-carbon enzyme, methylenetetrahydrofolate dehydrogenase2 (MTHFD2), which plays a key role in EBV-driven B cell growth and survival. MTHFD2 is essential for sustaining elevated NADPH pools in infected cells [24]. Paradoxically, mitochondrial NADPH oxidation via DHODH-coupled electron transport restricts excessive proliferation to avert metabolic exhaustion. This shows EBV’s precise balance of anabolic and redox needs during lymphomagenesis.

### EBV infection acts as a tumorigenic factor through epigenetic regulation

#### DNA methylation

EBV affects the epigenetic state of host cells and thus participates in the phenotypic changes of infected B cells. Upon establishment of latent infection, EBV promotes cell growth and metastasis through EBER1 and EBER2 non-coding RNAs, while EBNA1 disrupts the host response to DNA damage, these two mechanisms collectively induce ROS accumulation [37]. During the latent phase of EBV infection, quiescent B cells become proliferating lymphoblastoid cells expressing six EBV nuclear antigens and three latent proteins (LMP1, LMP2A, LMP2B). LMP2A, by methylating CpG islands, causes epigenetic alterations in the host genome and inactivates tumor suppressor genes, including PTEN and tumor-associated antigens [53]. Methylation of the viral genome not only alters the host genome, but also contributes to pathogen evasion of the host immune system. Other features of EBV-associated cancers include unique DNA methylation patterns, often referred to as the CpG island methylation phenotype (CIMP), which are seen in HL. These epigenetic alterations are caused by LMP-mediated overexpression of DNMT1 [54]. Meanwhile, EBNA3A and EBNA3C can exert epigenetic inhibitory effects by modifying DNA methylation at the promoter of the tumor-suppressor gene Bim, which in turn exerts epigenetic inhibitory effects [55].

B lymphocyte-induced maturation protein 1 (BLIMP1) exists in two major isoforms, a and b, which are produced by alternative promoters. Inactivation of the full-length isoform of BLIMP1a is proposed to promote B-cell lymphomas by blocking post-germinal center B (GCB) cell differentiation. Paul G. Murray et al. found that BLIMP1b expression increased during EBV infection of normal human tonsillar GCB cells. They also found that this expression change was accompanied by hypomethylation of the BLIMP1b-specific promoter. In this study, they demonstrated that the BLIMP1b promoter was hypomethylated in DLBCL cell lines, as well as in Hodgkin/Reed-Sternberg (HRS) cells of HL, suggesting that methylation modification of BLIMP1 may play a function in EBV-induced lymphoma [56].

#### Histone acetylation

The EBV latent antigens EBNA2 and EBNA-LP are the first latent genes to be expressed following B-cell infection [57]. EBNA2 is the major viral transcription factor that promotes B-cell proliferation by activating around 300 genes (MYC and RUNX3) [58, 59]. Importantly, this transcriptional activation is regulated by super-enhancers characterized by dense clustering of several transcription factors coupled with enhanced signals from the H3K27ac histone activation mark [60]. EBNA3C can form a complex with EBNA3A and EBNA3B [61]. Interacting molecules of EBNA3C include transcription factors, chromatin modulators (histone deacetylase and histone acetylase), post-translational modifiers, E3 ubiquitin ligases, and ubiquitin-specific proteases [62–66]. Similar to EBNA3C, EBNA3A interacts with many cellular proteins, such as members of the ubiquitin protease complex, chaperone proteins, and several proteins of unknown function associated with EBV-induced B-cell lymphomagenesis [64, 65]. These viral proteins lack specific binding-sequence similarity, yet their associated regions occasionally overlap functionally, suggesting a complex oncogenic mechanism where EBNA3A and EBNA3C act synergistically. Importantly, these binding regions are important during initial infection or maintenance of the growth of LCLs [67, 68]. EBNA3 proteins directly influence B-cell transformation and the development of B-cell lymphomas by targeting key cellular signaling cascades, including cell cycle, apoptosis, and autophagy. This includes direct protein-protein interactions, recruitment of chromatin remodeling factors (HAT, HDAC, and histone modifying enzymes), translational control (miRNAs), and protein degradation mechanisms (chaperones, proteases, and ubiquitin ligases) [64, 65]. EBNA3A and EBNA3C have been reported to inhibit p16 (INK4A) and p14 (ARF) by directly altering the levels of H3K27me3, H3K4me3, and H3 acetylation relative to quiescent B cells, albeit without altering CpG methylation [69].

#### Non-coding RNAs

In addition to nuclear and membrane-associated proteins, EBV expresses a variety of noncoding RNAs (ncRNAs), namely EBV-encoded nonpolyadenylated RNAs (EBER1 and EBER2), and numerous miRNAs during B cell infection [70]. Although most of these ncRNAs are not essential for B cell transformation, they contribute to immune evasion and are abundantly expressed. Overall, the role of EBERs in EBV-mediated B-cell transformation is somewhat contradictory [71]. Studies indicate that EBER1 and EBER2 are ncRNAs expressed in all forms of latency and are not essential for EBV-induced B lymphocyte transformation [72–74]. Another study found that expression of EBERs increased colony formation and induced B cell growth [75]. EBERs also interact with several important cellular partners. For example, the interaction of EBER1 with ribosomal protein L22 regulates protein translation, EBER1-mediated gene expression, and PKR-dependent apoptosis [75, 76]. The interaction of EBERs with RIG-1, AU-rich element-binding factor 1, and pattern recognition receptors activates host innate immune responses [75, 77]. In addition, EBER2 specifically recruits PAX5 to regulate LMP2A expression, as demonstrated by an EBER2 mutant virus with low LMP2A expression [78]. Meanwhile, EBER1 and several viral miRNAs are exported from infected cells via exosomes whose function correlates with the activity of surrounding cells [79].

The EBV genome encodes 44 mature miRNAs belonging to two distinct classes, BamHI-A region rightward transcription (BART) and BHRF1, which are expressed at different levels in different EBV latency types [80]. While BART miRNAs are expressed in almost all EBV-associated B-cell lymphomas, BHRF1 encodes miRNAs whose expression is relatively restricted to different latency procedures [81, 82]. Unexpectedly, these viral miRNAs regulate the expression of many cellular genes [83]. Moreover, viral miRNA expression levels were significantly higher in the early stages of infection than in transformed LCLs [84]. In addition to their crucial role in immune evasion during the early stages of B-cell viral infection, BART and BHRF1 miRNAs target a number of important cellular processes, especially those related to apoptosis and B-cell proliferation [83]. For example, BHRF1 miRNAs are required for B cell transformation by targeting multiple tumor suppressor proteins (PTEN and p27KIP1), whereas BART miRNAs block the expression of a variety of tumor suppressor genes (including DICE1, PUMA, PTEN, and BCL2L11), thereby promoting epithelial cell survival [85–88].

### EBV infection acts as a tumorigenic factor through altering the host immune microenvironment

**Fig. 2:**
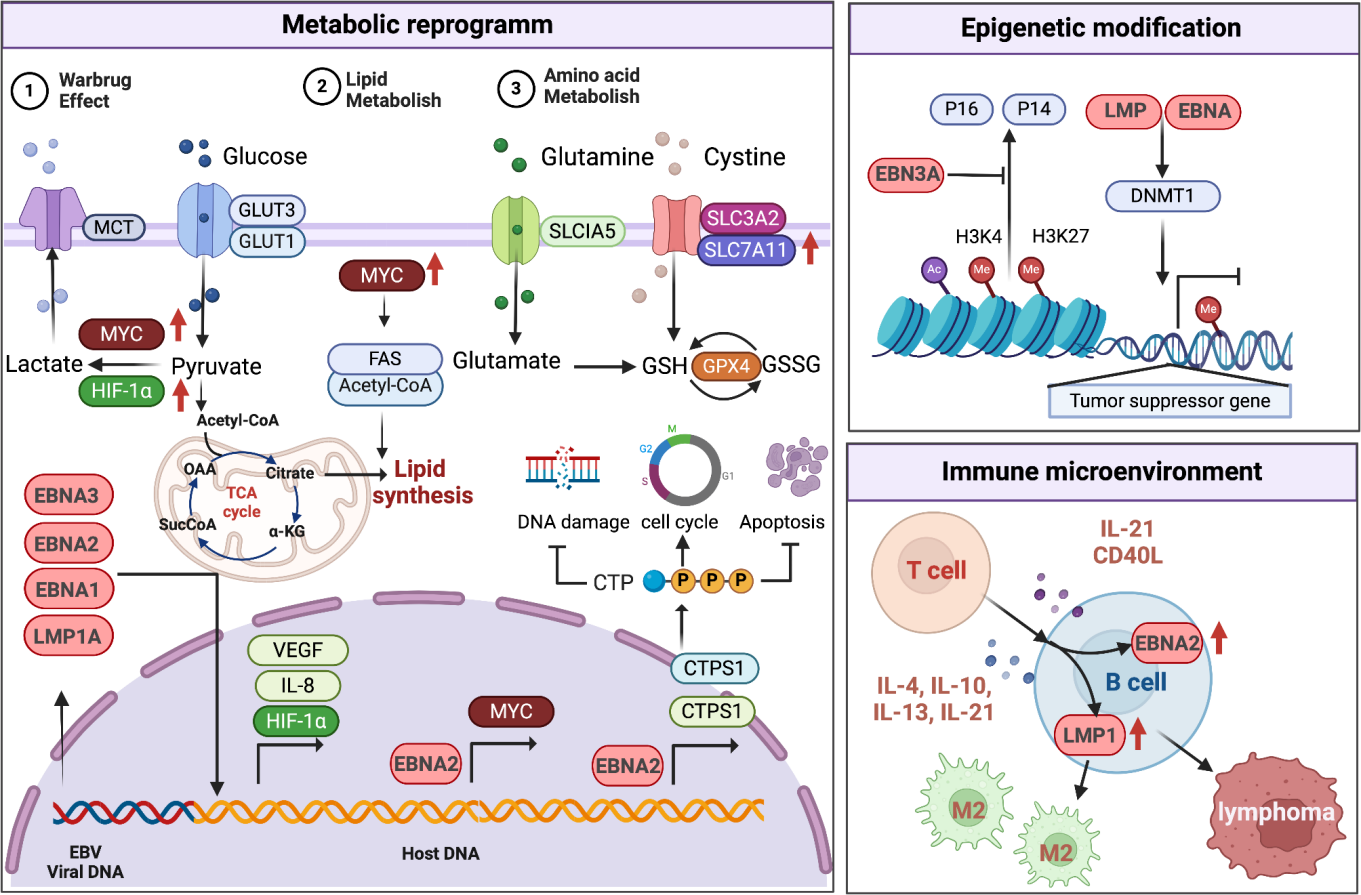
Molecular mechanisms of EBV oncogenesis in haematological malignancies. EBV infection acts as a tumorigenic factor through metabolic reprogramming, epigenetic modification, and immune microenvironment remodeling. This figure was created with BioRender.com

The expression of key EBV-encoded proteins is likely regulated by stromal-derived factors present in the cHL tumor microenvironment. In particular, the T-cell-derived cytokines IL-21 and CD40L down-regulate EBNA-2 expression in latent type III EBV-positive LCLs, whereas IL-4, IL-10, IL-13, and IL-21 induce LMP-1 expression in certain malignant B-cell lines. EBV can switch from latent to lytic infections, termed viral reactivation, which contributes to viral transmission and has the potential to cause a variety of diseases and complications. EBV reactivation can lead to uncontrolled B cell proliferation in immunocompromised individuals, including post-transplant lymphoproliferative disorders (PTLD) in recipients of hematopoietic stem cell transplantation (HSCT) or solid organ transplantation (SOT), and B-cell lymphomas in patients with acquired immune deficiency syndrome (AIDS) [89–91] (Fig. 2).

## KSHV/HHV-8

KSHV, also known as human herpesvirus 8 (HHV-8), primarily replicates and spreads by infecting endothelial cells and B lymphocytes. The transmission routes of KSHV include sexual contact, saliva, blood, and organ transplantation. KSHV is closely associated with diseases, such as Kaposi’s sarcoma (KS), KSHV-associated multicentric Castleman’s disease (MCD), primary effusion lymphoma (PEL), and KSHV-associated multicentric epithelial hyperplasia (MEH) [92].

### Viral proteins and infection mechanism

The KSHV genome consists of a central unique coding region of approximately 140 kb in length, flanked by non-coding terminal repeat units (TR) with high GC content, which are mainly responsible for viral protein expression [93]. Latency-associated nuclear antigen (LANA) is a key oncogenic protein of KSHV [94]. LANA is a multifunctional protein containing 1162 amino acids, 220–223 kDa in size. It is divided into an N-terminal, a C-terminal, and a central aspartate/glutamate repeats [95]. The C-terminal structural domain binds directly to conserved TR sequences of the KSHV genome. The chromatin-binding domain (CBD) in the lysine-rich N-terminus is docked to the host chromosome, thus forming an integral structure between the KSHV genome and the host chromosome [96].

The infection mechanism of KSHV involves several key steps: (1) attachment and entry: KSHV initially attaches to cells by binding to specific receptors on the host cell surface. These receptors include heparan sulfate proteoglycans (HSPGs), integrins (such as αVβ3 and αVβ5), and other receptors; (2) transport and maintenance of viral DNA: After entering host cells, the viral DNA is transported into the cell nucleus and maintained as a circular epigenetic structure known as an episome; (3) latent infection and lytic cycle: KSHV can establish latent infection within host cells, where only a few viral genes are expressed, which are enough to sustain viral replication and cell survival but not sufficient to trigger the immune system’s attention. Under certain conditions, the virus can enter the lytic cycle, expressing all viral genes, producing new virus particles, causing cell death, and releasing the virus; (4) virus-induced cell transformation: KSHV-encoded proteins can interfere with cell proliferation, inhibit apoptosis, and promote angiogenesis, collectively contributing to cell transformation and thus tumor formation.

### KSHV acts as a tumorigenic factor through metabolic reprogramming

#### Glucose metabolism

KSHV can induce aerobic glycolysis to support virus replication and promote the survival and proliferation of infected B cells. It has been reported that KSHV produces vFLIP and miRNA clusters, activating the NF-κB pathway, which suppresses glucose uptake by inhibiting glucose transporter 1 (GLUT1) and GLUT3 expression, thereby inhibiting oxidative phosphorylation [97]. Additionally, KSHV-encoded vGPCR and miRNA clusters upregulate HIF-1α expression, promoting aerobic glycolysis in B cells [98, 99]. Due to the abnormal over-proliferation of cancer cells, KSHV upregulates forkhead box protein O1 (FoxO1) and FoxO3, endowing tumor cells with strong antioxidant capabilities. This upregulation plays a crucial role in sustaining the progression of PEL, which is associated with the abnormal over-proliferation of cancer cells [100].

#### Amino acid metabolism

KSHV can enhance glutamine metabolism breakdown in normal cells, thereby inducing malignancy. LANA can stimulate B cells to upregulate glutaminase expression, and the latent protein Kaposin can stimulate the overexpression of metabotropic glutamate receptor 1 (mGluR1), enhancing glutamine breakdown metabolism in B cells and increasing their proliferation capacity [101]. KSHV can also increase the levels of GSH within B cells, enhancing their antioxidant stress capacity and inhibiting B cell apoptosis. Glutathione plays a crucial role in maintaining intracellular redox balance, and the expression of the amino acid transporter (xCT) on the cell membrane is essential for the uptake of cysteine needed for intracellular GSH synthesis [102]. Zhiqiang Qin et al. found abundant expression of xCT on the surface of KSHV^+^ PEL cells, which not only increases intracellular GSH levels to reduce oxidative damage caused by ROS but also activates the AKT pathway-dependent proliferation of B cells, ultimately promoting the progression of PEL. Additionally, he found that specific inhibitors of xCT, such as monosodium glutamate (MSG) and sulfasalazine (SASP), significantly increased apoptosis in KSHV^+^ PEL cells, suggesting that xCT may be a potential target for the treatment of KSHV^+^ PEL [103]. Recent studies have all indicated that KSHV infection induces metabolic reprogramming in B cells, which is crucial in driving the growth and survival of PEL cells.

### KSHV acts as a tumorigenic factor through epigenetic regulation

H3K27ac is typically localized at transcription start sites and enhancer regions, recruiting transcription factors and other transcriptional regulatory factors to promote chromatin structure openness and gene transcription activity [104]. During latent infection, the LANA protein generated can initiate enhancer-promoter activation and is associated with higher levels of H3K27ac, leading to the occurrence and development of PEL by activating the transcription of oncogenes CCND2 and MYC [105].

### KSHV induces tumorigenesis by altering the host immune microenvironment

**Fig. 3:**
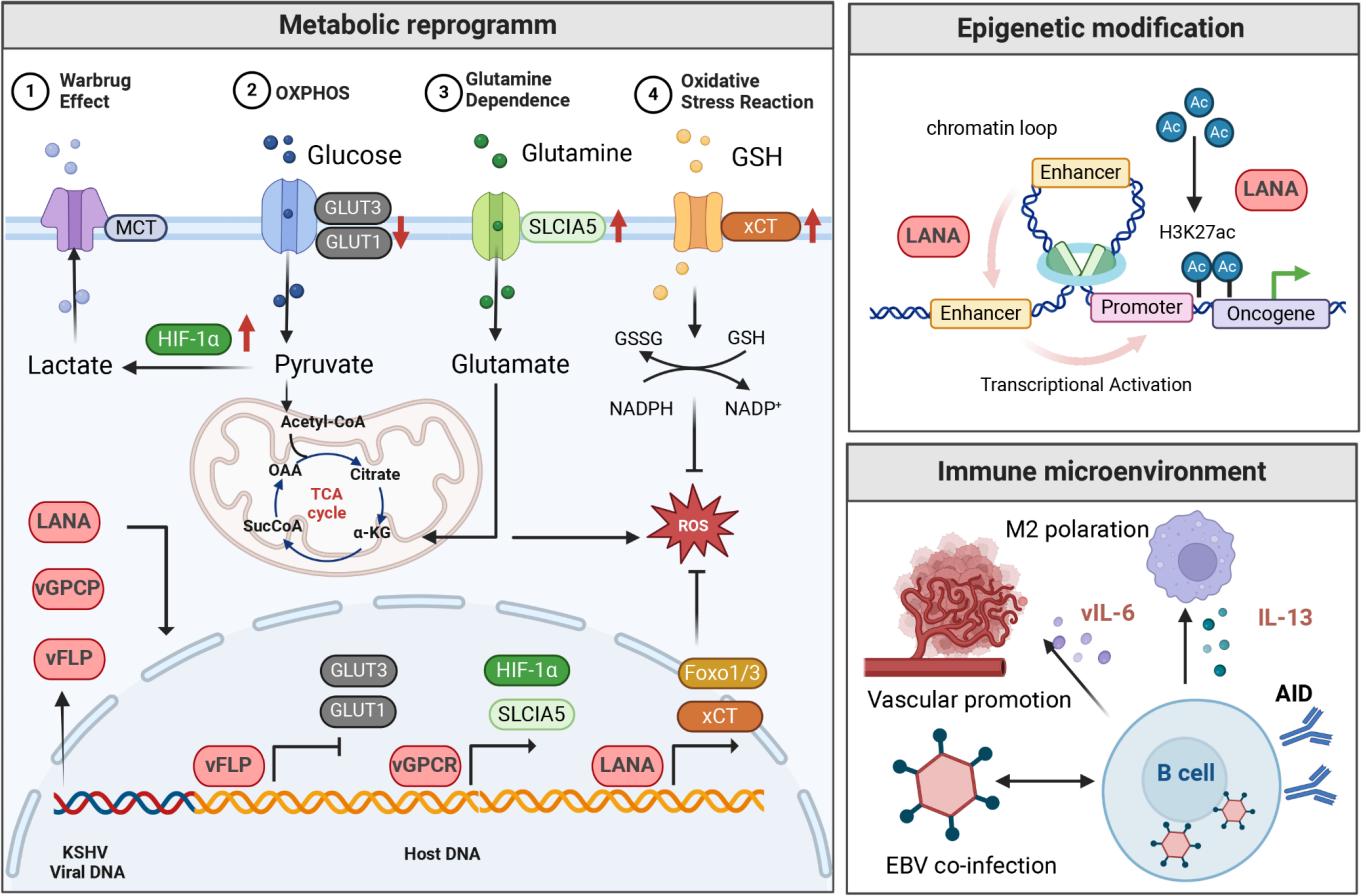
Molecular mechanisms of KSHV oncogenesis in haematological malignancies. KSHV infection acts as a tumorigenic factor through metabolic reprogramming, epigenetic modification, and immune microenvironment remodeling. This figure was created with BioRender.com

KSHV is associated with PEL, and 90% of KSHV^+^ PEL cases are also accompanied by EBV infection. Christian Münz et al. found that dual infection with EBV/KSHV enhances the persistence of KSHV and the occurrence of PEL [106]. The potential mechanism could be that KSHV persists in EBV-transformed B cells, promoting EBV lytic gene expression and leading to PEL formation. Münz et al. also found evidence of increased lytic EBV replication in lymphoproliferative diseases with human EBV/KSHV dual infection, further supporting this hypothesis [106]. Compared to other KSHV-related diseases, the levels of IL-13 secretion in the serum and effusions of PEL patients are significantly increased. Qiliang Cai et al. further demonstrated that IL-13 can activate the STAT6 pathway in KSHV^+^ PEL cells, promoting the growth and survival of PEL cells. Neutralizing antibodies against IL-13 significantly inhibit PEL cell proliferation and survival. Overall, these results suggest that IL-13/STAT6 signaling is modulated by KSHV to promote host cell proliferation and viral pathogenesis [107]. However, the molecular mechanisms by which KSHV infection upregulates IL-13 secretion in B cells require further exploration. Additionally, KSHV encodes a viral homolog of IL-6 called vIL-6. While IL-6 signals through the IL-6R and gp130 to activate downstream pathways, vIL-6 signals through gp130 homodimers [108]. Although both cytokines induce JAK-STAT signaling, promoting cell proliferation, angiogenesis, migration, and differentiation [109–111], vIL-6 also stimulates increased secretion of IL-6, leading to the progression of KS/PEL or the onset of multicentric Castleman disease (MCD), exacerbating malignant B cell proliferation [112]. The expression of vIL-6 at low levels in PEL cells contributes to cell proliferation and survival [113]. Furthermore, vIL-6 in PEL upregulates activation-induced cytidine deaminase (AID) expression in B cells, leading to increased class-switch recombination of IgG [114] (Fig. 3).

## HTLV-1

HTLV-1 is a retrovirus that infects human T lymphocytes, and is considered one of the etiological factors for adult T-cell leukemia/lymphoma (ATLL) and HTLV-1-associated myelopathy/tropical spastic paraparesis (HAM/TSP). HTLV-1 is primarily transmitted through blood contact, including sexual contact, mother-to-child transmission, and blood transfusion. After infection, HTLV-1 can remain latent in the host for long periods, with most infected individuals showing no apparent symptoms. However, 3–5% of infected individuals may develop diseases, such as ATLL or HAM/TSP several decades later, leading to serious health issues, such as malignancies and neurological disorders [115].

### Viral proteins and infection mechanism

HTLV-1 encodes several key viral proteins crucial for its replication, infectivity, and immune evasion. Tax is a pivotal transactivator protein that enhances viral gene expression and modulates cellular signaling pathways, promoting oncogenesis. Rex regulates the export of unspliced and singly spliced viral mRNA from the nucleus to the cytoplasm. Gag, Pol, and Env are structural proteins involved in virus assembly, reverse transcription, and entry into host cells, respectively. Additionally, HBZ (HTLV-1 bZIP factor) is expressed from the antisense strand and contributes to viral persistence and pathogenesis by modulating host gene expression and T-cell proliferation [116]. The infection mechanism of HTLV-1 involves the following key steps: (1) cell attachment and entry: HTLV-1 attaches to host cells by binding its glycoproteins on the viral envelope to specific receptors on the cell surface, facilitating cell attachment; (2) reverse transcription and integration: Once inside host cells, the RNA genome of HTLV-1 is transcribed by reverse transcriptase into double-stranded DNA. This newly synthesized viral DNA is then integrated into the host cell genome, becoming part of the cellular genetic material; (3) expression of viral genes: After integration into the host cell genome, genes of HTLV-1 begin to be expressed under the machinery of the host cell. It is worth mentioning that, in addition to possessing the classical structural features of retroviruses, the HTLV-1 genome also contains a region known as pX, which encodes regulatory proteins capable of producing Tax and bZIP factor (HBZ) [117]. (4) inter-cellular transmission: HTLV-1 primarily spreads through cell-to-cell contact, particularly by forming virological synapses or multinucleated cells, which transfer the virus from infected to uninfected cells. Following HTLV-1 infection, most individuals remain asymptomatic carriers for life, but a minority may progress to ATLL or HAM/TSP.

### HTLV-1 acts as a tumorigenic factor through metabolic reprogramming

#### Glucose metabolism

T cells undergoing malignant transformation often undergo a shift from oxidative metabolism to aerobic glycolysis, leading to a significant increase in glucose uptake, glycolysis, and subsequent lactate secretion [118]. Lactic acid, as the final product of aerobic glycolysis, can drive immune suppression and promote ATLL progression [119]. Charles R.M. Bangham et al. found that under different environmental pressures and inhibitors, physiological hypoxia (1% or 2% oxygen) can enhance HTLV-1 transcription. In contrast, inhibiting glycolysis or the mitochondrial electron transport chain suppresses plus-chain HTLV-1 transcription. This suggests that glycolysis promotes the reactivation of HTLV-1 [120]. Additionally, Robert Harrod et al. further revealed that HTLV-1 primarily promotes ATLL glycolytic metabolism through the p30II-p53 axis, inhibiting p53-K120 acetylation [121]. Other studies have shown that HTLV-1 not only drives T-cells towards aerobic glycolysis but also, through different mechanisms, activates the TAp73 promoter by its encoded HBZ RNA and HBZ protein, inducing the expression of MCT1 and MCT4, increasing lactate secretion, and inhibiting ATLL cell death [122].

#### Lipid metabolism

Seyed Abdolrahim Rezaee et al. conducted lipidomic analysis on individuals with HTLV-1-associated myelopathy/tropical spastic paraparesis (HAM/TSP) and HTLV-1 carriers, revealing a statistically significant positive correlation between the proviral load (PVL) in HTLV-1 carriers and cholesterol, triglycerides, and HDL levels. In HAM/TSP, cholesterol levels were found to be an independent risk factor for disease severity, suggesting that HTLV-1 can interfere with host cell lipid metabolism, thereby contributing to pathogenesis [123]. Additionally, other studies have found that HTLV-1 infection inhibits MSC adipogenic differentiation, thereby interfering with MSCs’ normal functions [124]. These studies collectively indicate that HTLV-1 can modulate host lipid metabolism. However, the impact of HTLV-1 on lipid metabolism in the ATLL population has not been reported, necessitating further research to gain insight into the effects of HTLV-1 infection on lipid metabolism in ATLL patients and explore its role in tumor progression.

### HTLV-1 infection acts as a tumorigenic factor through epigenetic regulation

Adult T-cell leukemia/lymphoma (ATLL) is a rare and aggressive T-cell lymphoma known for its elevated levels of H3K27me3. The H3K27me3 enzymes EZH1 and EZH2 are compensatory factors capable of stably regulating methylation patterns [125–127]. EZH2 is a histone modification factor frequently detected abnormally in cancer and alters the entire epigenome by increasing H3K27me3 levels. Targeting EZH2 has shown therapeutic efficacy in B-cell lymphomas and certain susceptible solid tumors [128, 129]. Seishi Ogawa et al. reported DNA methylation abnormalities of 53 genes, including those associated with apoptosis, providing new directions for drug development in ATLL [130].

### HTLV-1 infection acts as a tumorigenic factor by altering immune microenvironment

HTLV-1 receptors, such as GLUT1, neuropilin, and heparan sulfate proteoglycan are widely expressed in various cells, but the HTLV-1 retrovirus is primarily detected in CD4^+^ T cells in vivo [131, 132]. Furthermore, the immune phenotype of most HTLV-1-infected cells is characterized by CD45RO^+^ CD25^+^CCR4^+^CADM1^+^CD4^+^. In vitro experiments also indicate frequent upregulation of Foxp3 in HTLV-1-infected cells and ATLL cells, suggesting that HTLV-1 infection promotes the accumulation of Treg cells to construct an immunosuppressive microenvironment [133]. Since the HBZ protein is constitutively expressed in all HTLV-1-infected cells, it is speculated that the HBZ protein plays a crucial role in inducing Treg differentiation. Further in vivo and in vitro experiments confirm that the HBZ protein indeed induces the expression of immunosuppressive receptors, such as Foxp3, CCR4, and TIGIT [134, 135]. In addition to HBZ, other studies have reported that the HTLV-1-encoded Tax protein can attract and maintain a high-frequency circulation of CD4^+^Foxp3^+^CCR4^+^ Tregs [136]. These mechanisms favor the formation of an immunosuppressive microenvironment, reducing the efficiency of HTLV-1-specific CTL responses and thereby promoting the survival of ATLL cells [137]. However, it is intriguing that although 60–70% of ATLL cells express Foxp3, Foxp3^+^ HTLV-1-infected cells and Foxp3^+^ ATLL cells do not all possess Tregs’ suppressive functions [138–140]. The main reason is that after HBZ induces Foxp3 expression through Smad3-dependent TGF-β signaling [141]. Subsequently, it interferes with Foxp3’s DNA-binding activity and function through direct physical interaction [142]. The role of these cells induced by HTLV-1 to express Foxp3^+^ phenotype but lacking Treg function in the development of ATLL warrants further clarification.

**Fig. 4:**
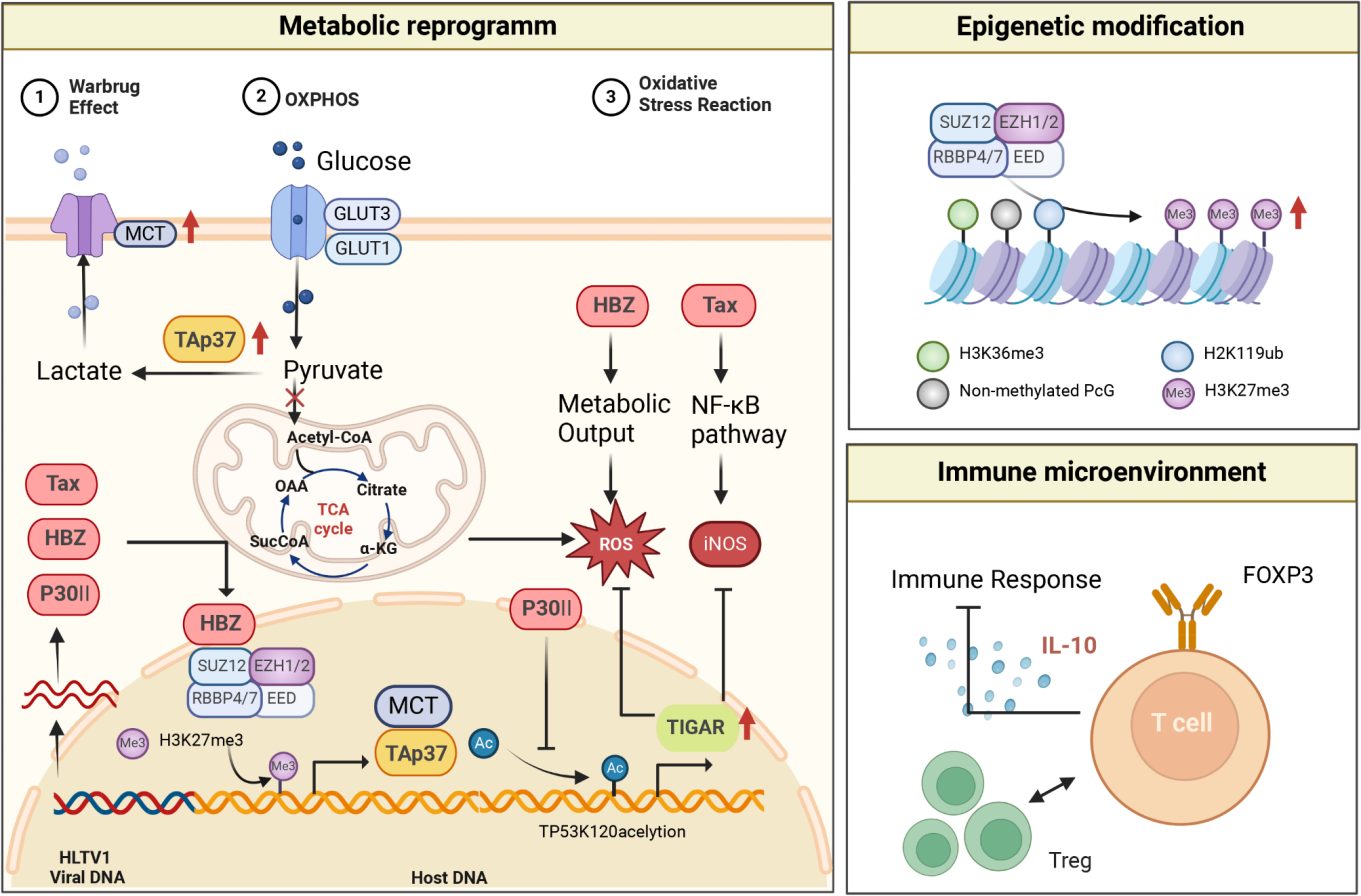
Molecular mechanisms of HTLV-1 oncogenesis in haematological malignancies. HTLV-1 infection acts as a tumorigenic factor through metabolic reprogramming, epigenetic modification, and immune microenvironment remodeling. This figure was created with BioRender.com

In addition to inducing the accumulation of Treg cells, HTLV-1 can also influence the secretion of cytokines in the microenvironment. Elevated levels of IL-10 have been observed in the serum of HTLV-1-infected and ATLL patients, with significant increases in IL-10 levels as the disease progresses [143, 144]. HBZ-transgenic mice revealed that TIGIT^+^CD4^+^ T cells are the primary source of elevated IL-10. By constructing HBZ-transgenic mice and detecting the secretion of factors from their TIGIT^+^CD4^+^ T cells, it was found that the increased IL-10 is mainly secreted by infected T cells [145]. IL-10 secreted by HTLV-1-infected cells inhibits host immunity via paracrine signaling while promoting ATLL cell proliferation via autocrine loops, demonstrating a double-faced role. Further studies have shown that after HTLV-1-infected cells secrete IL-10, it can inhibit host immune responses through paracrine pathways while simultaneously supporting the proliferation of ATLL cells through autocrine pathways, achieving a dual effect [146–148]. These studies suggest that excessive secretion of IL-10 may contribute to HTLV-1-infected cells evading host immune surveillance, exacerbating the development of ATLL (Fig. 4).

## HBV

HBV belongs to the family of hepatotropic viruses, and leads to liver cell damage, hepatitis, cirrhosis, and hepatocellular carcinoma. It mainly spreads through blood, sexual contact, and mother-to-child transmission. Some studies suggest a link between HBV infection and NHL. Literature reports indicate that the incidence rates of HBV and HCV in the NHL group were 3.3% and 1.3%, respectively, higher than those in the control group, suggesting an association between HBV or HCV infection and NHL [149]. Ke Zhuang et al. isolated cells with unlimited proliferative potential from the peripheral blood of four patients with chronic HBV infection, and these cells exhibited similar immunophenotype and biological behavior to most HBsAg-positive B-cell NHL patients, further supporting HBV-induced lymphoma development [150]. In addition to B-cell lymphoma and hepatocellular carcinoma, some studies suggest a possible association between HBV infection and the occurrence of other hematological malignancies, such as multiple myeloma. However, these associations still require further research for confirmation.

### Viral proteins and infection mechanism

HBV, a looped, partially closed, double-stranded DNA virus, encodes four major viral proteins: polymerase, envelope protein, core protein, and HBx protein [151]. HBx consists of 145–154 residues, lacks an X-ray crystal structure, and has the potential to form dimers. Positions C61, C69, and C137 of the HBx sequence are closely related to its transcriptional activation [152]. HBV infects hepatocytes through a specific interaction with the sodium taurocholate co-transporting polypeptide (NTCP) receptor on the hepatocyte surface. After binding, HBV enters cells via receptor-mediated endocytosis. Inside the cell, the viral envelope is removed, releasing the HBV genome—partially double-stranded DNA—into the nucleus. Here, the viral DNA is converted into covalently closed circular DNA (cccDNA), which serves as a template for transcription of the viral RNAs. These RNAs are translated into viral proteins and used to produce new viral particles that are released from the cell and can infect new hepatocytes, continuing the cycle [153, 154].

### HBV acts as a tumorigenic factor through metabolic reprogramming

Multiple studies have shown that persistent HBV infection reprograms host cell glucose metabolism, enhancing glycolysis, suppressing oxidative phosphorylation, and upregulating the pentose phosphate pathway. Enhanced glycolysis and suppressed oxidative phosphorylation play essential roles in promoting HBV replication and mediating HBV immune evasion [150, 155], while the pentose phosphate pathway protects cells from oxidative damage [156]. However, the phenomenon of HBV promoting aerobic glycolysis in host cells is mainly observed in hepatocellular carcinoma (HCC), and there is currently a lack of direct evidence regarding metabolic reprogramming in blood system tumors infected with HBV.

### HBV infection induces tumorigenesis through host epigenetic reprogramming

#### Non-coding RNAs

HBV can induce abnormal expression of lncRNAs in B cells, thereby mediating key processes in tumor development, invasion, metastasis, and chemoresistance [157, 158]. HBV-DNA consists of four segments (S, P, C, and X). HBx, encoded by the HBV-X segment, is considered a small “transcriptional activator” that stimulates transcription by activating the MAPK signaling pathway and relaxes cell cycle checkpoint control to promote cell proliferation, playing a role in virus-induced carcinogenesis [159, 160]. In HBV-positive DLBCL patients, it was found that HBx encoded by HBV can directly upregulate the expression of lncNBAT1 in DLBCL. After lncNBAT1 interacts with signal transducer and activator of transcription 1 (STAT1), it prevents the enrichment of STAT1 at the promoter region of apolipoprotein B mRNA editing enzyme catalytic subunit 3A (APOBEC3A), inhibiting the expression of APOBEC3A and enhancing DLBCL cells’ resistance to MTX [161].

Additionally, Alfonso Mele et al. found decreased expression of miR-34a in HBV^+^ B-NHL [162]. Previous studies have shown that downregulation of miR-34a affects HDAC activity and the NF-κB pathway, which may be closely related to B-cell proliferation and differentiation [163, 164]. It was found in the NB4 cell line that the use of HDAC inhibitors can promote the expression of miR-34a, inhibit cell proliferation, and induce cell apoptosis [165]. These studies all indicate that HBV downregulates miR-34a, leading to the malignant transformation of B cells, but the molecular pathways through which HBV regulates miR-34a and its specific molecular mechanisms affecting B cells are not fully understood.

#### Acetylation modification

Aberrant acetylation modification plays a significant role in HBV-related DLBCL patients. CREB-binding protein (CREBBP) mainly regulates histone acetyltransferase (HAT) activity, maintaining the activity of the HAT domain and intracellular acetylation levels [166]. HBV infection leads to a higher frequency of mutations in CREB-binding protein (CREBBP) in host cells [167]. Previous studies have shown that loss of function in CREBBP in human B cells results in localized depletion of enhancer H3K27 acetylation and aberrant transcriptional silencing of genes regulating B cell signaling and immune responses, thereby increasing the expression of BCL-6 and promoting the occurrence and development of B cell lymphoma [168]. SIRT1 also plays a significant role in the acetylation of B cell transcription factors. SIRT1 has been shown to deacetylate BCL-6, p53, and other transcription factors, upregulating BCL-6 expression [169, 170]. Recent research has found that chronic stimulation of HBsAg promotes the proliferation capability of human B lymphoblastoid cell lines through modulation of the SIRT1-NF-κB pathway [171].

### HBV induces tumorigenesis by altering the host immune microenvironment

During HBV infection, CTL cells are severely depleted, and remaining CD8^+^ and CD4^+^ T cells upregulate inhibitory receptors. When these receptors are cross-linked by ligands, T-cell functions are suppressed, leading to T-cell exhaustion and tumor escape [172]. Single-cell sequencing revealed that the GCB subtype in HBV-associated DLBCL was deficient in the expression of major histocompatibility complex II (MHC II) molecules and promoted tumor cell escape. In contrast, tumor cells of the ABC subtype, despite high expression of MHC II molecules, recruited more Tregs with potential immunosuppressive functions and could receive more reverse pro-proliferative signals from CD4^+^ T cells via the CD40-CD40LG axis, which in turn promoted the immune escape and growth of tumor cells [173, 174]. In addition, the microenvironment of HBV-associated DLBCL was significantly enriched with a subset of memory B cells that highly expressed the HBx target motifs EGR2 and EGR3. These cells are characterized by altered signaling pathways, homing, production of antiviral and pro-inflammatory cytokines (CD11c and CXCR3), and high expression of inhibitory receptors (PD-1), which not only affect the body’s ability to fight viruses, but also lead to immune escape of abnormal B cells [175, 176].

## HCV

HCV belongs to the Flaviviridae family, and its infection can lead to severe consequences, such as liver cell damage, chronic hepatitis, cirrhosis, and liver cancer. HCV primarily spreads through blood transmission, including blood transfusions, blood products, and sharing of injection equipment. Sexual contact and mother-to-child transmission are also routes of HCV transmission. While HCV mainly affects the liver, its infection may contribute to non-hepatic malignancies like NHL and lymphomas [177]. However, there is currently a lack of direct evidence for lipid metabolism reprogramming in hematological malignancies associated with HCV infection.

### Viral proteins and infection mechanism

The genome of HCV can be cleaved into ten different structural and non-structural proteins, among which HCV core protein (core), NS5a, and NS3 affect the function of host cells and are directly involved in the development of the disease process. The core protein, as a risk factor for HCV-related liver disease, play a crucial role in the interaction between the virus and the host cells and have direct oncogenic effects [178]. HCV primarily infects liver cells (hepatocytes). The infection process begins when HCV binds to specific receptors on the hepatocyte surface, including CD81, claudin-1, occludin, and scavenger receptor class B type I. After binding, the virus enters the cell through receptor-mediated endocytosis. Inside the cell, the HCV RNA genome is released into the cytoplasm, where it is translated and replicated using the host’s cellular machinery. The newly synthesized viral RNA and proteins are assembled into new viral particles that are released from the cell, mainly through a process involving the host’s lipid secretion pathways, to infect other cells [179].

### HCV acts as a tumorigenic factor through metabolic reprogramming

Lipid and nucleotide metabolism reprogramming is one of the primary metabolic alterations in cancer. This reprogramming plays a crucial role in cancer development [180]. Transcriptome sequencing revealed that HCV induces an increase in fatty acid and phospholipid synthesis in host cells, along with the inhibition of lipid degradation metabolism [181]. Peroxisome proliferator-activated receptors (PPARs) are nuclear receptor proteins, regulating lipid and lipoprotein metabolism. When they dimerize with retinoid X receptor (RXR), PPARs are activated. The activated complex promotes lipid clearance by upregulating β-oxidation [182]. The expression of HCV core protein can inhibit PPARα transcriptional activity, promoting cancer development [183]. Additionally, HCV core protein can enhance RXR transcriptional activity, leading to dysregulation of lipid metabolism enzymes and inducing HCC [177]. However, the upregulation of host cell lipid metabolism reprogramming by HCV is mainly observed in the HCC population, and there is currently a lack of direct evidence for the induction of hematological malignancies by HCV infection. Qiang Pan-Hammarström et al. integrated WGS/WES and lymphatic microarray data and found that 14 mutations were biased to occur in HBsAg-positive DLBCL patients, 11 of which were activation-induced AID potential off-target genes involved in cellular nucleotide metabolism. Mutational tag analysis identified tags associated with APOBEC, increasing the frequency of mutations in patients with HBV-associated DLBCL, and some of them were due to APOBEC/AID, suggesting that HBV may cause DLBCL through involvement in nucleotide metabolism [184].

### HCV infection induces tumorigenesis through host epigenetic reprogramming

Alfonso Mele et al. found abnormal levels of miR-223, miR-29a, and miR-29b in HCV^+^ splenic marginal zone lymphoma (SMZL). However, due to potential tissue-specific variations in miRNA levels associated with different histological subtypes of B-NHL, further studies with increased sample sizes and comparison of B-NHL tissues with the same histological classification are needed [162].

### HCV induces tumorigenesis by altering the host immune microenvironment

Studies have shown that HCV infection upregulates ferritin, TFR1, and DMT1 isoforms in macrophages, leading to increased iron accumulation within macrophages [185]. Accumulation of iron in macrophages polarizes them towards the M2 phenotype, reducing IL-15 expression, which can weaken T cell activation and drive HCV immune evasion [186].

## HCMV

HCMV is a herpesvirus that is widespread in the global population. HCMV infection is usually asymptomatic, but in individuals with compromised immune systems, such as those after organ transplants, or with AIDS. HCMV infection can lead to serious illnesses and complications. HCMV is transmitted primarily through saliva, blood, urine, breast milk and sexual contact. After infection, HCMV lurks in the host for life. Most people infected with HCMV have no noticeable symptoms, but when the immune system is compromised, it can lead to serious consequences, including organ transplant rejection, hepatitis, pneumonia, and encephalitis. Early views held that HCMV promotes cancer progression through oncomodulation by manipulating the tumor microenvironment, while emerging evidence showed the direct transforming effect of HCMV in several tumors [187, 188]. It has been reported that HCMV exerts a genuine oncogenic effect in glioblastoma, breast cancer, ovarian cancer, and prostate cancer [189–194]. Therefore, HCMV strains can lead to oncomodulation and oncogenesis.

### Viral proteins and infection mechanism

HCMV expresses several key viral proteins that facilitate its replication, immune evasion, and cell entry. Immediate-early proteins (IE1 and IE2) activate viral gene expression and modulate host immunity. PP65, a tegument protein, suppresses immune responses. Glycoproteins B, H, and L (gB, gH/gL) are essential for viral entry and fusion with host cells. UL97 kinase activates antiviral drugs and aids in replication. UL44 acts as a processivity factor for viral DNA polymerase. Proteins like US3, US6, and US11 interfere with antigen presentation, helping the virus evade immune detection. These proteins are crucial for HCMV’s life cycle and pathogenicity [195]. HCMV primarily targets epithelial and endothelial cells, where it binds to cellular receptors and enters via endocytosis or direct fusion of the viral envelope with the cell membrane. Inside the cell, HCMV releases its DNA into the nucleus, where it replicates using the host’s cellular machinery. The virus can establish a lifelong latent infection, remaining dormant in the body with periodic reactivations, especially when the host’s immune system is compromised. HCMV spreads between cells to avoid immune detection and enhance dissemination [196].

### HCMV acts as a tumorigenic factor through metabolic reprogramming

Many studies have confirmed that HCMV infection is involved in the development of type I diabetes mellitus (T1DM), and HCMV mainly attacks pancreatic islet cells directly or indirectly, thus reducing insulin secretion [197–199]. Unlike T1DM, in which pancreatic islet B-cells are severely damaged, the pathogenesis of T2DM is somewhat different, and islet cell function is still present in T2DM patients. Existing studies have also been limited to the high prevalence of HCMV infection in patients with T2DM [200]. Meanwhile, viperin (endoplasmic reticulum-associated interferon-induced viral inhibitory protein), which is directly induced by HCMV, may be involved in lipid and glucose metabolism by interacting with HCMV mitochondrial inhibitor of apoptosis (vMIA) proteins [201, 202]. While HCMV disrupts cellular glucose-lipid metabolism, the impact of this disruption on hematological oncogenesis has not yet been reported.

### HCMV acts as a tumorigenic factor through epigenetic regulation

Joseph L. Wiemels et al. found that congenital HCMV infection is a risk factor for childhood ALL by detecting the presence of HCMV sequences in newborn blood spots [203]. Evidence indicates that HCMV infection induces acute lymphoblastic leukemia (ALL) by histone modification. Recent work demonstrates that HCMV infection upregulates EZH2 and activates Myc, driving trimethylation of H3K27 and silencing tumor suppressor genes [204]. This synergy between viral-induced EZH2 hyperactivity and Myc dysregulation may represent a key mechanism in HCMV-mediated cellular transformation.

HCMV exploits miRNA networks as a key epigenetic strategy to drive leukemogenesis in ALL. HCMV induces distinct miR-155/miR-92 dysregulation patterns in ALL, compared with T-ALL. B-ALL patients commonly show elevated miR-155 and suppressed miR-92 expression. This is often linked to HCMV infection, with HCMV potentially disrupting CD8⁺ T cell differentiation programs [205]. Notably, HCMV seropositivity correlates with TNF-α-mediated miR-155 overexpression in the B-cell population [206], while viral epigenetic reprogramming of the miR-17-92 cluster is mechanistically linked to B-lymphomagenesis [207, 208]. These coordinated miRNA perturbations exemplify HCMV’s tumorigenic plasticity through epigenetic hijacking.

### HCMV induces tumorigenesis by altering the host immune microenvironment

Although HCMV may not directly cause cancer, it is known to have a wide-ranging impact on immune function, including in utero, where many pre-leukemic ALL clones begin. In fetuses infected with HCMV, there is a significant increase in the percentage of activated and terminally differentiated CD8^+^ T cells in umbilical cord blood, including both HCMV-specific and nonspecific T cells [209]. Following HCMV infection, fetal γδ T cells proliferate and differentiate [210]. HCMV-induced “adaptive” CD94/NKG2C^+^ NK cells are also increased in preterm infants infected postnatally with HCMV and in children with a history of congenital HCMV infection [211]. One study showed that compared to infants not infected with HCMV, those infected had increased B-cell proliferation [212], and the expression of B-cell activation genes in infants infected with HCMV was higher than in healthy controls [213]. While B-cell activation and antibody production may be beneficial for anti-pathogen immune responses, premature activation of precursor B cells could be detrimental. HCMV retains a range of extensive immune evasion strategies that can modulate immune surveillance and ALL risk. HCMV interferes with antigen presentation by proteasomal degradation of MHC class I heavy chains [214]. NK-mediated cytotoxicity in HCMV-infected cells is limited by the viral peptide UL40, which mimics HLA-C and inhibits NK function [215]. Antibody-mediated immunity is also compromised in utero and postnatally [216]. A study on 524 children with ALL found HCMV in 44% of the children’s ALL blast samples [217]. Compared to children in whom HCMV was not detected in tumor progenitor cells, children with HCMV-positive ALL had more frequent infections in the first six and twelve months of life. Although these were not confirmed as congenital HCMV infections, they indicate that HCMV-associated immune dysregulation in childhood is related to the increased frequency of symptomatic childhood infections, which could contribute to leukemia development. Compared to HCMV-negative cases, cytokine signaling pathways, including IL-1, IL-7, IL-8, and the B-cell receptor signaling pathway, are upregulated in the bone marrow of children with HCMV-positive ALL cases. Furthermore, in HCMV cases, pathways involving Th1 cells and those responsible for crosstalk between dendritic cells and NK cells are downregulated. IL-7, a cytokine involved in the development of B-ALL, may propose a potential mechanism for the pathogenesis of B-cell leukemia by HCMV [218]. Children developing ALL show cytokine production dysregulation at birth [219], and exposure to HCMV proteins in utero is related to cytokine patterns associated with ALL risk [220].

In HCMV-seropositive adults, HCMV-specific T cells account for 10–50% of the total memory T cell pool. Congenital HCMV also affects the developing immune system, including components of T, NK, and B cells [211, 221]. Meanwhile, HCMV impacts the T-cell pool through massive amplification of the virus-specific memory pool and expansion of terminally differentiated T cells/effectors. This may impair the immune response to new antigens in the elderly and the quantity of CD8^+^ memory T lymphocytes. The substantial formation and maintenance of HCMV-specific CD8^+^ T cells, known as “memory inflation”, are mechanisms of immunosenescence [222]. Although HCMV is the most significant virus affecting T lymphocyte dysregulation, there are few studies analyzing the hematological pathological impact of HCMV, likely because the primary effect of HCMV is to cause T lymphocyte dysfunction. T-cell lymphomas are very rare. Seeking causality in more common B-cell lymphomas includes looking for indirect reasons caused by the fundamental relationships between T lymphocytes and B lymphocytes [223].

## MPyV and HPV

Merkel cell polyomavirus (MCPyV) is a small circular DNA virus isolated from Merkel cell carcinoma (MCC) [224, 225]. MCPyV has significant genomic homology with the African green monkey lymphotropic polyomavirus that infects B lymphocytes, prompting speculation that MCPyV may be associated with human B-cell lymphoma [224]. In addition, epidemiologic studies have shown that the risk of chronic lymphocytic leukemia/small lymphocytic lymphoma (CLL/SLL) is 30 times higher in patients with MCC [226]. Eric J. Duncavage et al. found that MCPyV was present in lymphocytes in a small percentage of CLL/SLL cases. HPV is a common DNA virus primarily associated with the development of cervical, penile, vulvar, vaginal, and oropharyngeal cancers [227, 228]. HPV infections are usually asymptomatic. There is currently no direct evidence of an association between HPV and hematological tumorigenesis. The virus-driven carcinogenesis is summarized in Table 2.

**Table 2:** Summary of virus-driven carcinogenesis

Name of Virus	Target cell	Diagnostic molecule	Hematological malignancy subtypes	Common Features
EBV	B cell	EBV-encoded small RNA	eBL/sBL DLBCL HL PCNSL PEL	EBER positive Frequent tumor-infiltrating lymphocytes EBER positive
HIV	CD4^+^ T cell	p24	HL NHL PEL	Immune exhaustion
HTLV-1	CD4^+^ T cell	ATLL antibody	ATLL	Frequent CCR4 mutation
KSHV	B cell	Latency-associated nuclear antigen	PEL	LANA positive
MCV	CD8^+^ T cell	CM2B4	ALL	CM2B4 positive
HBV	B cell	HBs antigen	NHL	Frequent TP53 mutation
HCV	B cell	anti-HCV antibody	NHL	Frequent TERT mutation Possible regression by viral elimination
HPV	B cell	p16	NA	p16 positive

eBL, endemic Burkitt lymphoma; sBL, sporadic Burkitt lymphoma; DLBCL, diffuse large B-cell lymphoma; HL, Hodgkin lymphoma; PCNSL, primary central nervous system lymphoma; PEL, primary effusion lymphoma; NHL, non-Hodgkin lymphoma; PEL, primary effusion lymphoma; ATLL, adult T-cell leukemia/lymphoma; EBER, EBV-encoded small RNAs; NA, not applicable

## HIV

HIV belongs to the family of retroviruses and destroys the host’s immune system by infecting specific cells in the human immune system, especially CD4^+^ T lymphocytes, resulting in impaired immune functions. HIV infections are usually categorized into two main types: HIV-1 and HIV-2, with HIV-1 being the most common strain responsible for the majority of AIDS cases globally. HIV is primarily transmitted through blood, sexual contact, mother-to-child transmission, and sharing of injection equipment. Its infection may lead to HL, NHL, KS, and PEL [229].

### Viral proteins and infection mechanism

**Fig. 5:**
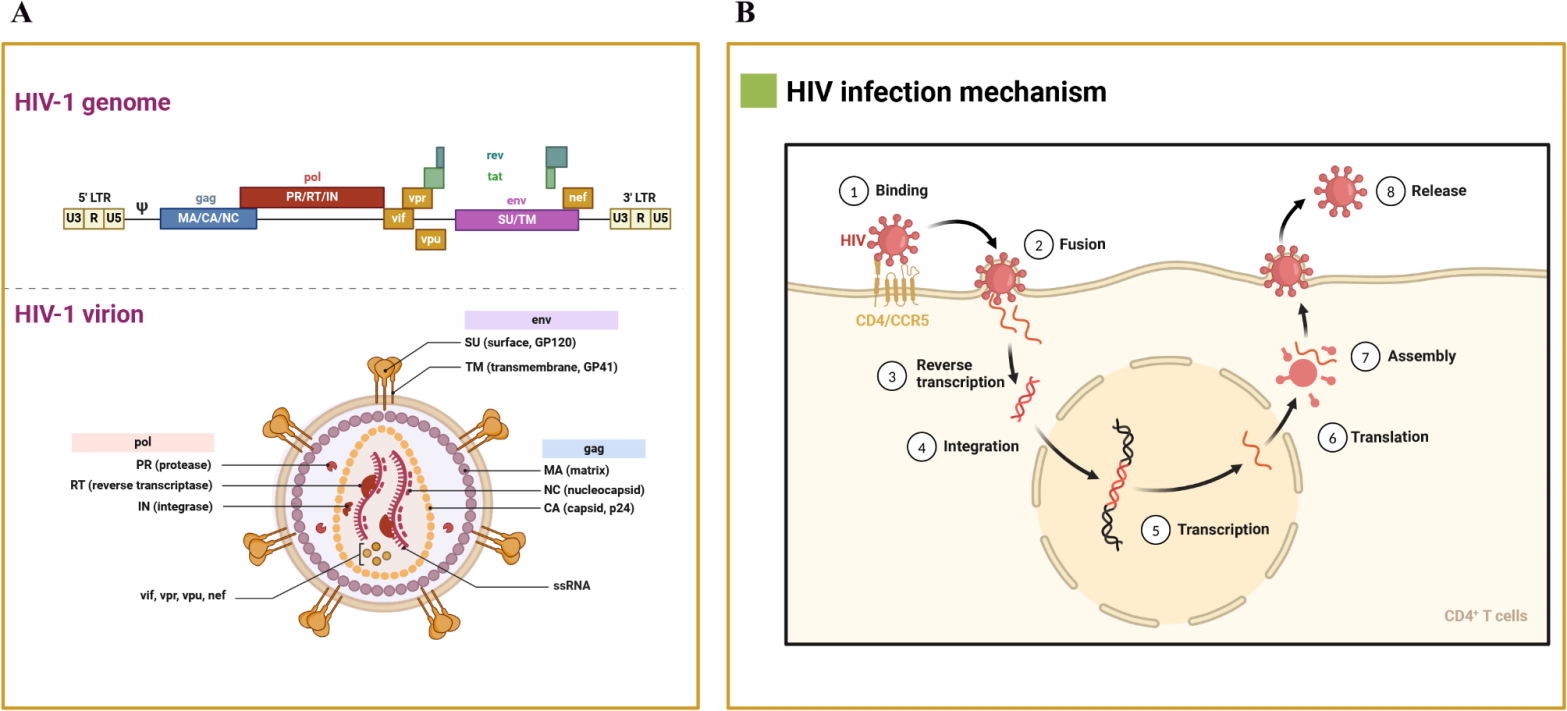
HIV-1 genome and infection mechanism. (**A**) HIV is an enveloped virus encoding three polyproteins (Gag, Pol, and Env) and six accessory proteins (Tat, Rev, Nef, Vpr, Vif, and Vpu); (**B**) HIV invades host cells and replicates its genetic material through a complex series of steps, a process that involves binding, fusion, DNA synthesis, integration, and the production of new virus particles using host cell surface receptors. This figure was created with BioRender.com

HIV is an enveloped virus containing a 9.8 kb positive-sense RNA genome encoding three polyproteins (Gag, Pol, and Env) and six accessory proteins (Tat, Rev, Nef, Vpr, Vif, and Vpu) [230]. HIV invades host cells via receptor-mediated binding and fusion. Once inside, it reverse-transcribes its RNA into DNA, integrates the DNA into the host genome, and uses host cell machinery to produce new virus particles. (1) binding and fusion: The surface glycoprotein gp120 of the HIV virus recognizes and binds to the CD4 receptor and its coreceptors, such as CCR5 or CXCR4. Subsequently, fusion of the virus with the cell membrane occurs, allowing viral RNA, reverse transcriptase, integrase, and other viral proteins to enter the host cell; (2) reverse transcription: viral RNA is reverse transcribed into double-stranded DNA, a process catalyzed by the virus-carrying reverse transcriptase enzyme; (3) integration: newly synthesized viral DNA is transported to the nucleus and embedded in the host cell’s DNA by integrase; (4) translation and assembly: the host produces new viral proteins and RNA, which are subsequently assembled into new viral particles within the cell; (5) release: the newly formed viral particles are released from the host cell by cytotoxicity, which in turn infect other cells (Fig. 5).

### HIV infection acts as a tumorigenic factor through metabolic reprogramming

#### Glucose metabolism

Mitochondria are crucial in providing adenosine triphosphate (ATP) to meet the energy demands of the cell, and their dysfunction can lead to cell death [231], as seen in the case of HIV/SIV infection [232]. In T cells, the ligation of the costimulatory receptor CD28 can induce the expression of the Bcl-xL transcription factor, which controls the levels of glucose uptake and glycolysis [233]. Glucose is one of the primary carbon sources for lymphocytes, supporting bioenergetics and biosynthesis; restricting the concentration of this nutrient results in reduced cell activation and proliferation. HIV-1 glycoprotein gp120 stimulates glycolysis [234]. Increased glycolysis, also known as the Warburg effect, is a well-known feature of most tumors that supports unrestrained proliferation and invasion of tumor cells [235, 236]. Gp120 promotes tumor cell proliferation, migration and survival when expressed in viral particles, on the surface of infected cells, or as a virus-free soluble protein [234, 237].

#### Lipid metabolism

Lipid metabolism refers to the mechanisms involved in the synthesis, storage, and breakdown of lipids. Fatty acid synthesis (FAS) starts with acetyl-CoA carboxylase (ACC) carboxylating acetyl-CoA to malonyl-CoA [238, 239]. On the other hand, fatty acid oxidation (FAO) is the process where fatty acids are converted into acetyl-CoA to produce ATP. Changes in lipid metabolism during HIV infection have been extensively reported. In ART-naïve individuals, the expression of peroxisome proliferator-activated receptor-γ (PPAR-γ) and lipogenic genes is lower, such as lipoprotein lipase and fatty acid-binding protein 4 (FABP4), which are essential for lipogenesis and lipid storage [240]. HIV replication alone is known to induce the production of free fatty acids, triglycerides, and FASN [241]. Moreover, in ART-naïve HIV patients, the levels of FAO products like laurate, myristate, palmitate, and stearate are reduced and not normalized by ART treatment, indicating a decrease in FAO during HIV infection [242]. John R. Koethe et al. found that expansion of memory CD4^+^ T cells in adipose tissue may contribute to alterations in adipocyte energy storage and metabolism, and ultimately to the development of diabetes. This phenomenon may be amplified in the HIV population, a group with a disproportionately high prevalence of metabolic diseases [243]. While the disturbances in lipid metabolism caused by HIV itself and ART treatment are well established, there is less research on how HIV might induce hematological malignancies through lipid metabolic processes, warranting further in-depth studies.

#### Amino acid metabolism

Untreated HIV infection elevates IDO activity and Trp degradation, correlating with increased Tregs and Th17/Treg imbalance, whereas ART restores these parameters [244]. HIV elite controllers (ECs) exhibit a unique tryptophan degradation metabolism, where low levels of tryptophan accumulation in plasma, rather than Kynurenine (Kyn), are associated with unchanged Th17/Treg balance [244]. Most recent studies suggest that in both pretreatment and post-ART viral suppression, IDO activity (measured by Kyn/Trp ratio) positively correlates with the size of the HIV-DNA reservoir [245]. Among amino acid transport proteins, the xCT-antitransporter protein consists of xCT (also known as SLC7A11) and its chaperone CD98, which acts as a Na^+^-independent electroneutral exchange system for cystine/glutamate [246]. Expression of xCT on the cell membrane is essential for the uptake of cystine, which is required for intracellular glutathione (GSH) synthesis and plays an important role in maintaining intracellular redox homeostasis [102, 247]. Targeting xCT-induced caspase-dependent apoptosis, the xCT-selective inhibitor sulfasalazine (SASP) blocked PEL tumor progression in vivo [103].

### HIV infection acts as a tumorigenic factor through epigenetic regulation

#### DNA methylation

DNA methylation is critical for transcriptional regulation in HIV-1 latency [248], and HIV-1 infection also increases de novo methylation of genes, such as p16 (INK4A) in lymphoid cells [249]. HIV proviral DNA is subject to epigenetic regulation through enzyme complexes, which control the DNA’s interaction with these histones through costimulatory modification of the amino-terminal ends of the core histones and ATP-dependent enzyme complexes [250]. CpG methylation also controls the reactivation of latent HIV [251]. The above studies suggest that epigenetic mechanisms are involved in the HIV-1 cycle. HIV-1 has also been shown to influence other components of the host epigenetic machinery. For example, the HIV-1 Tat protein inhibits SIRT1 deacetylase and induces T-cell hyperactivation [252]. Shotaro Hagiwara et al. found 2541 targets to be significantly different in HIV^+^ lymphomas by DNA methylation microarray analysis. Laminin, collagen, N-cadherin, and caveolin2, which are associated with cell adhesion, were significantly hypomethylated in HIV-associated lymphomas, suggesting that their increased expression triggers and promotes tumors, leading to a poor prognosis [253–256]. Hypomethylated FGF5, a bone metastasis-associated gene associated with angiogenesis [257], may similarly influence the prognosis of HIV-associated lymphomas [258].

#### Histone acetylation and methylation

Epigenetic transcriptional processes drive HIV latency in T cells, as the repressive state of chromatin contributes largely to HIV latency [259]. Qin Feng et al. found that H3K27 acetylation specifically recruited the super elongation complex (SEC), a transcriptional elongation complex necessary for HIV-1 long terminal repeat sequence (LTR)-mediated and general cellular transcription. Interestingly, H3K27 acetylation further stimulates H3R26 methylation, which in turn eliminates SEC recruitment, creating a negative feedback regulatory loop. Importantly, HIV transcription was reactivated in several HIV latency cell models by inhibiting the methyltransferase activity of CARM1, the enzyme responsible for H3R26 methylation. The above studies suggest that coordinated and dynamic modifications of histones H3K27 and H3R26 can orchestrate HIV LTR-mediated transcription and may open a new way to disrupt latent HIV infection by targeting specific epigenetic enzymes [260]. Despite the recognition that epigenetic mechanisms are involved in HIV-1 latency, there is currently no available data on the effects of HIV integration or reactivation on cellular epigenomic profiles.

#### Non-coding RNAs

HIV and its components regulate the expression of host miRNAs, which play an important role in tumorigenesis [261]. HsamiR-200c-3p is significantly down-regulated in HIV-associated BL, and the zinc-finger E-box-binding homology cassette epithelial-mesenchymal transition (EMT) transcription factors ZEB1 and ZEB2 are up-regulated, which actively promote tumor metastasis and invasion. In addition, miR-21 was significantly elevated in peripheral B cells from people living with HIV (PLWH), suggesting that it may contribute to maintaining B cell hyperactivation [262]. Several studies conducted on B-cell lymphomas have shown upregulation of the miR-17-92 cluster. This upregulation in lymphomas leads to the inhibition of p21 (p21 is a CKI, hence a regulator of cell cycle progression in G1 and S phase), thereby contributing to the development of B-cell lymphomas [263]. A study found that overexpression of miR-17-92 is a common feature in all HRLs, regardless of their germinal center or non-germinal center origin [264]. Furthermore, this aberration is associated with inhibition of p21 in BL and DLBCL, further supporting the role of miRs in the pathogenesis of HRLs. MiR-21 is the most commonly overexpressed miRNA in cancer. It is highly expressed in hematological malignancies, such as CLL [265], DLBCL [266, 267], acute myeloid leukemia (AML) [266], and HL [268]. Compared to HIV-negative controls, miR-21 is significantly elevated in peripheral B cells of PLWH progressing to NHL. Additionally, miR-21 is overexpressed in activated B cells and can be induced by IL-4 alone or in combination with CD40 or IgM co-stimulation with lipopolysaccharide, suggesting that miR-21 may contribute to maintaining excessive activation of B cells and promoting lymphomagenesis [262]. These studies indicate that miRNAs are important contributing factors in the pathogenesis of HALs.

### HIV infection acts as a tumorigenic factor by altering host immune microenvironment

While HIV sequences have not been detected in malignant transformed B cells, the impact of viral infection diminishes immune surveillance and allows the escape of transformed lymphoid cells. In this scenario, EBV and HHV-8 infections trigger excessive production of B cells and increase the risk of genetic alterations leading to lymphoma [269]. Deposition of HIV-1 in the oropharynx [270], anal/rectal region [271], cervicovaginal area, foreskin/penile region [114, 270, 272, 273], airways [274], and gastric epithelial cells promote systemic infection of CD4^+^ T lymphocytes, Langerhans/dendritic cells, and macrophages both in vivo and in vitro [114, 272, 275–279]. This “contamination” creates a pro-inflammatory environment characterized by the production of inflammatory mediators, such as IL-6 and TNF-a, and impairs cell adhesion [274, 277, 280]. Specifically, the HIV-1 matrix protein p17 can create a prelymphatic vessel-generating microenvironment, facilitating lymphoma growth and metastasis in lymph nodes [281]. Meanwhile, NK cells exhibit functional impairment, compromised cytotoxic function, altered cytokine secretion, and impaired antibody-dependent cellular cytotoxicity. Indeed, HIV-induced loss of NK cell-mediated control of lytic EBV infection has been explicitly shown to contribute to lymphomagenesis and also enhance HCMV replication [282].

**Fig. 6:**
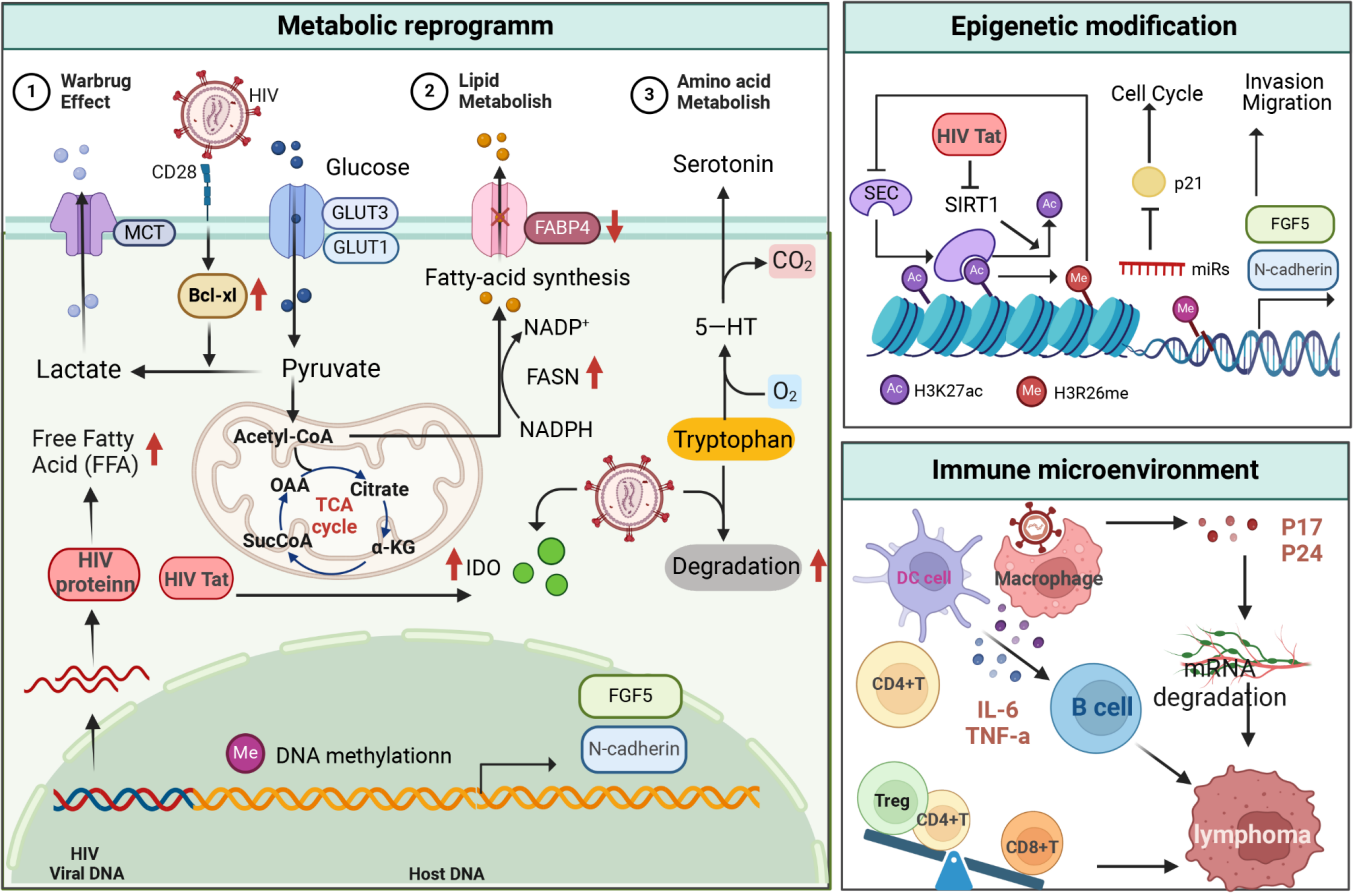
Molecular mechanisms of HIV oncogenesis in haematological malignancies. HIV infection acts as a tumorigenic factor through metabolic reprogramming, epigenetic modification, and immune microenvironment remodeling. This figure was created with BioRender.com

Macrophages serve as the primary reservoir for HIV. They are implicated in the progression of HIV-associated lymphomas (HALs). Exposure to IL-4 and IL-10 activates tumor-associated TAMs into M2 macrophages. These activated M2 macrophages promote tumor angiogenesis [283]. Additionally, in HIV-related DLBCL, there is a significant reduction in helper CD4^+^ T cells and FOXP3^+^ regulatory T cells. Cytotoxic T cells, especially activated CD8^+^ T cells, are significantly increased in these DLBCL cases. The increase in CD8^+^ T cells may be associated with the local presence of LMP-1 (EBV antigen) and p24 (HIV antigen) [284]. Soluble molecules, such as cytokines (IL-1, IL-2, IL-6, IL-10) and chemokines (CCL and CXCL families) also play crucial roles in HAL development. HIV infection disrupts cytokine levels; this dysregulation of cytokine pathways plays a significant role in lymphoma development [285]. Huysentruyt et al. found that 60% of HALs had intracellular staining of the p24 protein in macrophages, suggesting that these infected cells may participate in the development of these tumors through pro-inflammatory activity [283] (Fig. 6).

## Treatment of virus-associated hematological malignancies

### Vaccination strategies

Prophylactic vaccines targeting oncogenic viruses have transformed cancer prevention, with HBV and HPV vaccines demonstrating remarkable success. Universal HBV vaccination has reduced hepatocellular carcinoma incidence by 72% in endemic regions through neonatal immunization programs [286]. HPV vaccines show > 90% efficacy against high-risk genotypes linked to lymphoid malignancies, though male vaccination rates remain suboptimal despite rising oropharyngeal cancer risks [287]. In contrast, vaccine development for EBV and KSHV faces biological hurdles: EBV nanoparticle vaccines targeting gp350/gH/gL achieved seroconversion but limited protection against lymphomagenesis [288], while KSHV’s antigenic variability complicates epitope selection despite promising mRNA-based prototypes [289]. Therapeutic vaccines complement prevention efforts. HBV formulations combining PreS1/S antigens with TLR9 agonists yield 32% functional cure rates when paired with antivirals, and adenovirus-delivered mosaic antigens induce transient T-cell responses against HTLV-1 oncoproteins [290]. Emerging technologies address these limitations through structural vaccinology and neoantigen discovery via single-cell profiling of virus-associated tumors [291, 292]. These advances underscore the need for tiered strategies combining prevention, therapeutic augmentation, and precision antigen design.

### Antiviral therapies

Effective management of virus-associated hematological malignancies often begins with antiviral therapies that specifically target the viral infections contributing to tumor development [293, 294]. For instance, antiviral drugs like nucleoside analogues have been effective against EBV and HBV, reducing viral load and thereby decreasing the oncogenic potential of these viruses. Additionally, antiretroviral therapy (ART) for HIV not only controls the virus but also significantly reduces the risk of developing HALs. Ongoing research aims to enhance the efficacy of these treatments and to discover specific antiviral agents for viruses like HTLV-1 and KSHV, where direct antiviral treatment options are currently limited. Nucleotide analogues (NA) and interferons are the two types of antiviral drugs currently approved for the treatment of HBV infection. NA, including entecavir, tenofovir disoproxil fumarate, and tenofovir alafenamide, inhibit viral replication by inhibiting reverse transcription of pre-genomic RNAs in the cytoplasm to HBV DNA. Recent studies have shown that entecavir in combination with chemotherapy improves the prognosis of patients with HBV-associated DLBCL [295].

### Metabolism therapies

Currently, numerous metabolism-modulating drugs are undergoing evaluation in clinical trials, with some already approved by the FDA for cancer treatment. It is hoped that combining them with conventional chemotherapy and the latest targeted therapies will enhance anti-tumor efficacy while suppressing treatment toxicity [296]. Metformin, the most commonly used drug for the treatment of type 2 diabetes, has been shown to have anticancer properties. Mara Cirone et al. found that metformin activated AMPK in KSHV^+^ PEL and down-regulated its pro-survival pathways, such as mTOR and STAT3, which triggered PEL apoptosis. Metformin enhanced bortezomib-mediated cytotoxicity against PEL cells and inhibited the activation of the KSHV cleavage cycle, suggesting that metformin alone or in combination with bortezomib is a promising strategy for PEL [297].

### Epigenetic therapy

Understanding epigenetic changes in cancer has led to therapies targeting epigenetic regulators, a method known as epigenetic therapy [298–300]. Several epigenetic drugs, including DNA demethylating agents and histone deacetylase (HDAC) inhibitors, have now been FDA-approved for cancer treatment. Suberohydroxamic acid (SBHA) is a selective HDAC inhibitor, mainly targeting HDAC1 and HDAC3, which can play an anti-growth role in many malignant tumors, including breast cancer. Currently, SBHA has been shown to induce histone acetylation in the promoter region of KSHV replication and transcription activation genes, and induce apoptosis in PEL cells by inducing acetylation and phosphorylation of p53, with dual therapeutic effects on KSHV^+^ PEL [301]. Many drugs are still in the early stages of trial, and their efficacy and usability in NKT lymphoma patients still need to be further considered [302–304].

### Immunotherapy

The role of the immune system in controlling or eradicating virus-induced tumors is pivotal. Immune checkpoint inhibitors, which have revolutionized the treatment of various cancers, are being studied for their efficacy in treating hematological malignancies with viral etiologies [305]. These therapies may help restore immune function that is often suppressed or evaded by oncogenic viruses. Additionally, therapeutic vaccines to enhance the immune response against oncogenic viruses and virus-infected cells are under development. For example, therapeutic vaccines targeting EBV antigens in nasopharyngeal carcinoma are undergoing clinical trials and could be adapted for EBV-associated hematological malignancies [306].

### Combination therapies

Combining antiviral therapy with immunomodulatory agents and epigenetic therapy can be more effective than single-agent treatments. This approach not only aims to eradicate the virus but also to tackle the complex tumor microenvironment that supports cancer progression. Clinical trials exploring the efficacy of such combination therapies in treating virus-associated hematologic malignancies are critical and may bring new hope for patients, and the present clinical trial of virus-driven hematological malignancies is shown in Table 3.

**Table 3:** Clinical trials of virus-driven haematological malignancies

Lymphoma subtype	Treatment regimen	Outcomes	NCT number
EBV^+^ lymphoma	EBV-CTLs Brentuximab Vedotine Sintilimab + R-CHOP Nanatinostat + valaganciclovir mRNA vaccine Tislelizumab + R-CHOP TCR-T cell	CR:68%, one-year OS:88.9% ORR: 48%, Median OS:15.6 momths NA ORR:40%, Median OS:10 months NA NA NA	NCT01498484 NCT02388490 NCT04181489 NCT05011058 ChiCTR2200065962 ChiCTR2100054451 ChiCTR2100050405
HIV^+^lymphoma	Vorinostat + R-DA-EPOCH Peripheral blood stem cells Brentuximab Vedotine + AVD RNA interference + allo-HSCT anti-CD20 iNK-T cells/anti-CD19/CD22 CAR-T cells	ORR:100%, 1-year OS:83% NA OS:92%,2-year PFS:95% NA NA	NCT01193842 NCT00002221 NCT01771107 ChiCTR2300076061 ChiCTR2000028826
HTLV-1^+^ lymphoma	NA	NA	NA
KSHV^+^lymphoma	Oral Azacitidine Lenalidomide + R2-EPOCH Nivolumab + Varlilumab	NA Two-year OS: 66.7% NA	NCT04799275 NCT02911142 NCT03038672
MCV^+^lymphoma	NA	NA	NA
HBV^+^lymphoma	Entecavir + Tenofovir /Entecavir Ibrutinib Tenofovir Alafenamide + R-CHOP Chidamide Chidamide	NA NA NA NA NA	NCT04539119 NCT02991638 NCT03804372 NCT04661943 ChiCTR1800017698
HCV^+^lymphoma	Sofosbuvir + ledipasvir/ribavirin Sofosbuvir + Velpatasvir/Ledipasvir IFN/PegIFN with ribavirin Interferon-free antiviral treatment	NA ORR:45%, three-year PFS:80% NA NA	NCT02717949 NCT02836925 NCT03261349 NCT02762448

NA, not applicable; CTLs, cytotoxic T lymphocytes; CR, complete response; OS, overall survival; ORR, objective response rate; PFS, progression-free survival; TCR, T-cell receptor; CAR-T cell, chimeric antigen receptor-T cell; allo-HSCT, allogeneic hematopoietic stem cell transplantation; AVD: doxorubicin, vinblastine, and dacarbazine; R-CHOP: rituximab, cyclophosphamide, doxorubicin, vincristine, and prednisone; R-DA-EPOCH: dose-adjusted etoposide, prednisone, vincristine sulfate, cyclophosphamide, doxorubicin hydrochloride and rituximab; R2-EPOCH: lenalidomide in combination with R-DA-EPOCH

## Limitations of this review

This review also has some limitations. First, although the review covers a range of important oncogenic viruses, it might not include all viruses known or suspected to be linked with hematological malignancies. The exclusion of other less studied or newly discovered oncogenic viruses could limit the comprehensiveness of the findings. In addition, the potential interactions between different oncogenic viruses and their cumulative or synergistic effects on hematological malignancies have not been discussed in this review, but understanding these interactions could be important for a holistic view of viral oncogenesis. Finally, the review has not addressed geographic or demographic variations in the prevalence of viruses and how these factors influence the incidence and type of hematological malignancies. Such insights are crucial for developing targeted strategies in diverse populations.

## Conclusions and perspectives

This review has comprehensively examined the significant role of various oncogenic viruses, including EBV, HIV, HTLV-1, KSHV, HCMV, HBV, and HCV in the pathogenesis of hematological malignancies. It is well-established that these viruses contribute to tumor development not only by directly interfering with cellular functions but also by modulating metabolic pathways and epigenetic landscapes, thereby reshaping the immune microenvironment to favor both viral replication and tumor progression. Such insights are crucial for understanding the multifaceted nature of virus-associated tumorigenesis and underscore the importance of targeting these viruses in therapeutic strategies. Further investigations into the specific molecular mechanisms through which these viruses exert their effects are essential. This includes a deeper exploration into the interplay between viral proteins and host cell targets, particularly how these interactions lead to persistent infection and immune escape. Additionally, the role of newly discovered or less commonly studied viruses in hematological malignancies warrants more attention, as they may reveal novel pathways of disease pathogenesis.

Current treatment approaches for oncogenic viruses associated with hematological malignancy include vaccination strategies, antiviral therapies, and emerging metabolism-targeting agents and epigenetic therapies. Meanwhile, immunotherapies leveraging checkpoint inhibitors or virus-specific CAR-T cells directly enhance anti-tumor immunity. Combination regimens integrating these modalities show particular promise in overcoming resistance mechanisms. Further investigations must prioritize: (1) Optimizing therapeutic vaccines against herpesviruses using mRNA platforms; (2) Defining biomarkers for metabolic therapy responsiveness in virus-driven malignant niches; (3) Resolving immunotherapy resistance mechanisms in immunosuppressive viral microenvironments. Integrating virologic and oncologic targeting through these multimodal approaches offers transformative potential for precision oncology.

## Data Availability

No datasets were generated or analysed during the current study.
